# Supplementary material for: Genetic nurture in intergenerational transmission of substance use
Source: Nat Commun. 2026 Mar 26;17:4446. doi: 10.1038/s41467-026-71175-8 (PMC13183951; doi:10.1038/s41467-026-71175-8)
Supplement: Supplementary file 1 — Supplementary Information [file 41467_2026_71175_MOESM1_ESM.pdf]

SUPPLEMENTARY INFORMATION

Genetic Nurture in Intergenerational Transmission of Substance Use

Authors: Mannan Luo, Victória Trindade Pons, Nathan A. Gillespie, Hanna M. van Loo

SUPPLEMENTARY METHODS

Participants

This study included a total of 19,233 genotyped adult offspring who had at least one parent genotyped, comprising 15,966 parent-offspring pairs and 3,267 complete mother-father-offspring trios. For the analysis of overall genetic nurture effects using mixed-effect regression modeling, the sample size varied depending on the availability of substance use data, with a maximum of 15,863 (13,411 parent-offspring pairs and 2,452 complete trios). For parent-of-origin and mediation analyses using structural equation modeling (SEM), full-information maximum-likelihood estimation (FIML) was applied to handle missing data[1]. This approach enabled the inclusion of all 19,233 genotyped adult offspring.

Quality control and imputation of genotype data

Lifelines participants were genotyped using three different arrays: the Illumina CytoSNP-12v2 array, the Infinium Global Screening Array® (GSA) MultiEthnic Disease Version 1.0, and the FinnGen Thermo Fisher Axiom® custom array, in order of release. Quality control (QC) of marker and samples were performed separately per array. For the CytoSNP array (released in 2020), quality control (QC) involved filtering SNPs with a minor allele frequency (MAF) above 0.001, a Hardy-Weinberg equilibrium (HWE) p-value >1e-4. A call rate threshold of 0.95 was used for both markers and samples. Sample QC included principal component analysis (PCA) to detect

population outliers, and removal of duplicates, individuals with high heterozygosity and ambiguous sex, resulting in 249,249 markers and 15,422 samples. The UMCG Genetics Lifelines Initiative (UGLI) release 1 underwent a two-step QC for marker and sample missingness thresholds (from <80% to <99%), removing monomorphic markers (MAF = 0) and those with HWE p-value >1e-6. Samples with heterozygosity >4 standard deviations from the mean, duplicates, and ambiguous sex were excluded, yielding 548,029 markers and 36,339 samples. The UGLI release 2 (Affymetrix array) followed a similar two-step QC for call rates (first <80%, then <99%), removing markers with HWE p-value >1e-10 and MAF <0.02, and excluding samples with heterozygosity >4 SD from the mean, duplicates, and those with sex or family discrepancies, resulting in 462,731 markers and 28,249 samples. All arrays were imputed using the Haplotype Reference Consortium (HRC) panel (http://www.haplotype-reference-consortium.org) via the Sanger Imputation Service (http://imputation. sanger.ac.uk). Population stratification was examined using PCA with samples from the 1000 Genomes Project, retaining only individuals of European ancestry. Post-imputation, we filtered for imputation quality (INFO >0.8) and MAF >0.05 and selected high-quality markers (HapMap3+) in each array. We then selected markers that were available in all three arrays, resulting in 1,161,061 common markers. This overlap allowed for reliable haplotype comparison across parent-offspring pairs in the sample of 19,235 offspring with at least one genotyped parent, before matching with phenotype data. Detailed QC reports for each array are available on the Lifelines wiki (http://wiki-lifelines.web.rug.nl/).

Polygenic scores (PGS) imputation

For regression models, imputation non-transmitted PGS were performed similarly to the approach described in Kong et al.[2]. For parent-offspring trios and pairs, parental transmitted PGS

(PGS_T) were computed as the sum of the transmitted maternal and paternal haplotypic PGS. Parental non-transmitted PGS (PGS_{NT}) were calculated by summing the scores from both parents. In parent-offspring pairs where data for one parent was missing, the missing PGS_{NT} value for that parent was imputed using the mean score of all available scores from parents of the same type (e.g., the mean paternal PGS was used if the father's data was missing, and the mean maternal PGS if the mother's data was missing) before summing the scores.

Statistical analysis

Longitudinal associations between polygenic scores and offspring cigarettes per day

Equation 1: Main effects model

$$Current\ CPD_{ij} \sim PGS_{Ti} + PGS_{NTi} + age_{ij} + sex + birth\ year + waves + (1 + age \,||\, individual\ ID) + (1|\,Family\ ID) + \varepsilon_{ij}$$

Equation 1. Linear mixed-effects model examining main effects of polygenic scores on offspring cigarettes per day (CPD). Subscript *i* denotes the individual, the *j* denotes the timepoint. Fixed effects include transmitted polygenic score (PGS_T), non-transmitted polygenic score (PGS_{NT}), age at measurement, sex, birth year, and data collection wave. The coefficients for PGS_T and PGS_{NT} represent the primary parameters of interest, estimating average direct genetic effects and genetic nurture effects on current CPD over time, respectively. Random effects include individual-specific random intercepts and random slopes for age (1 + age || individual ID) to account for between-person differences in baseline CPD and age-related change. Family-specific random intercepts (1 | Family ID) account for sibling relatedness. The residual error is denoted by ε_{ij}.

Equation 2: Age interaction model

$$Current\ CPD_{ij} \sim PGS_{Ti} \times age_{ij} + PGS_{NTi} \times age_{ij} + age_{ij} + sex + birth\ year + waves + (1 + age \,||\, individual\ ID) + (1|\,Family\ ID) + \varepsilon_{ij}$$

Equation 2. Linear mixed-effects model examining age-moderated effects of polygenic scores on offspring cigarettes per day (CPD). This model extends Equation 1 by including interaction terms between age and both polygenic scores (PGS_T × age and PGS_{NT} × age). The interaction coefficients represent the primary parameters of interest, testing whether direct genetic effects and genetic nurture effects change as individuals age. Random effect structure is identical to Equation 1.

SUPPLEMENTARY REFERENCES

1 Enders, C. K. *Applied missing data analysis*. (Guilford press, 2010).

2 Kong, A. *et al.* The nature of nurture: Effects of parental genotypes. *Science* **359**, 424-428 (2018). https://doi.org/doi:10.1126/science.aan6877

SUPPLEMENTARY TABLES

All statistical tests were two-sided unless otherwise specified.

Supplementary Table 1. Sensitivity analyses: regression estimates for transmitted (PGS_T) and non-transmitted (PGS_{NT}) polygenic scores for cigarettes per day predicting smoking quantity in combined, pair-only, and trio-only samples.

Outcome	Analysis	*N*	PGS_{T_CPD}		PGS_{NT_CPD}	
			β (95% CI)	*p*	*β* (95% CI)	*p*
CPD	Combined	5,972	.202 (.177, .226)	<.001	.037 (.012, .062)	.004
	Pair-only	5,134	.195 (.169, .222)	<.001	.042 (.013, .071)	.004
	Trio-only	838	.243 (.177, .309)	<.001	.023 (-.027, .072)	.368
Pack-years	Combined	6,276	.136 (.114, .158)	<.001	.028 (.006, .050)	.014
	Pair-only	5,382	.132 (.109, .156)	<.001	.027 (.002, .053)	.037
	Trio-only	894	.162 (.104, .221)	<.001	.030 (-.014, .074)	.184

Note. Estimates are from linear mixed-effects regression models including up to *N* = 6,276 adult offspring with at least one parent genotyped and available data on smoking outcomes. Models adjusted for age and sex and included a family-ID random intercept to account for sibling clustering. CPD = cigarettes per day; *β* = standardized regression coefficient; CI = confidence interval, *p* = unadjusted *p* value. No adjustments were made for multiple comparisons, as these sensitivity analyses assess consistency of the main findings across subsamples.

Supplementary Table 2. Regression estimates of transmitted and non-transmitted polygenic scores for substance use disorder with offspring substance use outcomes

		PGS_{T_SUD}				PGS_{NT_SUD}			
Outcomes	*N*	β_T (SE)/OR	95% CI	*p*	p_{FDR}	β_{NT} (SE)/OR	95% CI	*p*	p_{FDR}
Smoking initiation	15853	1.340	1.285, 1.398	<.001	<.001	1.002	.962, 1.042	.932	.970
Cigarettes per day	5972	.115 (.013)	.091, .141	<.001	<.001	.021 (.013)	-.005, .045	.111	.278
Pack-years	6276	.101 (.011)	.079, .123	<.001	<.001	.021 (.011)	-.002, .042	.070	.278
Daily alcohol intake	15863	.079 (.008)	.064, .094	<.001	<.001	.003 (.008)	-.012, .018	.719	.970
Cannabis initiation	9097	1.161	1.084, 1.243	<.001	<.001	.998	.935, 1.067	.970	.970

Note. Smoking initiation and cannabis initiation were modeled with logistic mixed-effects models (OR shown). Cigarettes per day, pack-years, and daily alcohol intake were modeled with linear mixed-effects models (β and SE shown). All models adjusted for age and sex and included a family-ID random intercept to account for sibling relatedness. *N* indicates the number of offspring with available outcome data and at least one genotyped parent. β = standardized regression coefficient; OR = odds ratio; CI = confidence interval; SUD = substance use disorder; *p* = unadjusted *p* value; p_{FDR} = *p* value after false discovery rate (FDR) correction for multiple comparisons.

Supplementary Table 3. Covariance estimates of maternal and paternal polygenic scores in the parent-of-origin effects analysis

	Maternal PGS_{T_CPD}			Maternal PGS_{NT_CPD}		
	β (SE)	95% CI	p	β (SE)	95% CI	p
Paternal PGS_{T_CPD}	.006 (.007)	-.009, .019	.421	.012 (.009)	-.006, .029	.171
Paternal PGS_{NT_CPD}	.006 (.010)	-.014, .025	.570	-.005 (.017)	-.040, .026	.753

Note. Structural equation modeling was used to test parent-of-origin effects, while accounting for covariances between maternal and paternal transmitted ad non-transmitted polygenic score due to potential genetic assortative mating on smoking. As the analyses used a full information maximum likelihood (FIML) approach to handle missing data, retaining all available data from the full genotyped family sample (N = 19,233), there was no list-wise N for the sample. Standardized coefficients (β), standard errors (SE), 95% confidence intervals (CIs) and unadjusted p value are reported.

Supplementary Table 4. Monte Carlo power analysis for transmitted and non-transmitted parent-of-origin effects on smoking quantity

Outcome		Transmitted		Non-transmitted	
	CI Level	Effect (*Δβ*)	Power	Effect (*Δβ*)	Power
CPD	95% CI Lower	-.067	.971	-.045	.478
	Observed	-.032	.447	.003	.051
	95% CI Upper	.003	.057	.050	.567
Pack-years	95% CI Lower	-.056	.955	-.050	.686
	Observed	-.025	.371	-.008	.064
	95% CI Upper	.006	.074	.035	.376

Note. Statistical power was estimated from Monte Carlo simulations (1,000 replications) to evaluate statistical power across the range of plausible effect sizes defined by our observed estimates and their 95% confidence intervals. Simulations preserved key empirical data features: N = 19,233 individuals from 13,649 families, data missingness patterns handled via full-information maximum likelihood, and empirical predictor correlations. Evaluated effect $\Delta\beta$ denotes the value of the parent-of-origin contrast (maternal-paternal).

Supplementary Table 5. Parent-of-origin effect contrasts in mediation and proportion mediated effects

Mediator	Effect type	*Δβ*	95% CI	*p*
Cigarettes per day	Mediated effect (transmitted)	.018	.006, .029	.003
	Mediated effect (non-transmitted)	.022	.010, .033	<.001
	Proportion mediated (transmitted)	.157	.063, .251	.001
	Proportion mediated (non-transmitted)	.542	-.151, 1.234	.125
Packyears	Mediated effect (transmitted)	.012	.004, .020	.004
	Mediated effect (non-transmitted)	.016	.007, .025	<.001
	Proportion mediated (transmitted)	.148	.061, .268	.004
	Proportion mediated (non-transmitted)	.704	-4.023, 6.015	.884

Note. Mediation analysis using structural equation modeling: maternal/paternal smoking quantity as a mediator of the associations between transmitted (PGS_T) and non-transmitted (PGS_{NT}) polygenic scores and offspring smoking outcomes. The full information maximum likelihood (FIML) was applied to leverage all available data from the full genotyped family sample (*N* = 19,233). All models adjust for offspring sex and ages. Parent-of-origin effects in mediation were tested across two distinct aspects: (i) the absolute size of the mediated pathway via parental smoking, and (ii) the proportion of the total effect explained by parental smoking. *Δβ* denotes the absolute value of the parent-of-origin contrast (|maternal - paternal|), with 95% bootstrap confidence intervals. *β* = standardized coefficient; CI = confidence interval; *p* = unadjusted p value. No adjustments were made for multiple comparisons as the parent-of-origin contrasts were pre-specified primary tests within a single mediation model, and inference relied on bootstrap confidence intervals for the mediation effects.

Supplementary Table 6. Multi-group mediation analysis: sex differences in parental transmitted and non-transmitted pathways via parental cigarette per day

Mediated effects via parental CPD			
Pathway	Parent	Daughters *β* (95% CI)	Sons *β* (95% CI)
Transmitted	Maternal	.028 (.018, .039)	.022 (.010, .036)
	Paternal	.009 (.001, .019)	.010 (.000, .021)
Non-Transmitted	Maternal	.030 (.020, .041)	.025 (.012, .041)
	Paternal	.007 (.001, .015)	.009 (.000, .020)
Within-sex comparison			
Pathway	Contrast	Daughters *Δβ* (95% CI)	Sons *Δβ* (95% CI)
Transmitted	Maternal - Paternal	.019 (.003, .034)	.013 (-.008, .033)
Non-Transmitted	Maternal - Paternal	.023 (.008, .038)	.017 (-.004, .036)
Between-sex comparison			
Pathway	Contrast	Maternal *Δβ* (95% CI)	Paternal *Δβ* (95% CI)
Transmitted	Daughters - Sons	.006 (-.011, .022)	-.001 (-.016, .014)
Non-Transmitted	Daughters - Sons	.005 (-.014, .022)	-.002 (-.015, .011)

Note: A multi-group structural equation modeling (SEM) mediation analysis stratified by offspring sex was conducted to simultaneously estimate maternal and paternal mediation pathways for daughters and sons. Within-sex comparisons contrast maternal versus paternal pathways; between-sex comparisons test whether mediation effects differ between daughters and sons. The analysis included 15,871 offspring (9,827 female, 6,044 male) with at least one genotyped parent, nested within 13,347 families. CPD = cigarettes per day. Effects are presented as standardized coefficients with 95% confidence intervals; intervals not crossing zero indicate statistical significance. No adjustments were made for multiple comparisons because all mediation pathways were pre-specified and estimated within a single multi-group model.

Supplementary Table 7. Summary statistics of genome-wide association studies used to calculate polygenic scores

Phenotype	Abbrev.	N_{Total}	Number of independent SNPs	SNP heritability (SE)	Citation
Smoking Initiation	SmkInit	2,669,029	1,752	.08 (.002)	Saunders et al. 2023
Cigarettes Per Day	CPD	618,489	153	.08 (.005)	Saunders et al. 2023
Drinks Per Week	DPW	2,428,851	501	.04 (.001)	Saunders et al. 2023
Cannabis Initiation	CanInit	184,765	8	.11 (.01)	Pasman et al. 2018
Substance use disorders	SUD	1,025,550	19	NA	Hatoum et al. 2023

Supplementary Table 8. Longitudinal associations between transmitted and non-transmitted polygenic scores and current cigarettes per day across adulthood

Predictor	Model 1: Main effects		Model 2: Age interactions	
	β (95% CI)	*p* value	β (95% CI)	*p* value
Fixed effects				
PGS_{T_CPD}	.115 (.092, .138)	<.001	.120 (.097, .143)	<.001
PGS_{NT_CPD}	.026 (.003, .049)	.029	.026 (.003, .049)	.028
Age	-.040 (-.054, -.024)	<.001	-.040 (-.054, -.025)	<.001
Sex (male)	.085 (.039, .132)	<.001	.085 (.039, .132)	<.001
Birth year	-.248 (-.376, -.120)	<.001	-.249 (-.377, -.121)	<.001
Wave 2	-.016 (-.101, .068)	.704	-.016 (-.101, .068)	.705
Wave 3	.024 (-.124, .171)	.752	.024 (-.123, .172)	.746
Interaction terms				
PGS_{T_CPD} × Age			-.0035 (-.0058, -.0013)	.002
PGS_{NT_CPD} × Age			-.0004 (-.0027, .0020)	.761

Note. Results from linear mixed-effects models examining the association between polygenic scores and current cigarettes per day (CPD), with repeated measures within individuals (*n* = 6,885) nested within families (*n* = 5,755), totaling 9,725 observations across three waves. Age was centered at the baseline (wave 1) mean (33.0 years) to facilitate interpretation. The main effects model tests whether transmitted (PGS_{T_CPD}) and non-transmitted (PGS_{NT_CPD}) polygenic scores for CPD, irrespective of time, are associated with offspring current CPD across adulthood. The interaction model includes PGS_{T_CPD} × Age and PGS_{NT_CPD} × Age terms to test whether these associations change with age. All models adjusted for age, sex, birth year, and measurement wave. We additionally included random intercepts and slopes for individuals to account for repeated measures, and random intercepts for families to account for sibling relatedness. β = standardized coefficient, 95% CI = 95% confidence interval. Model comparison confirmed that including PGS × age interactions significantly improved model fit (likelihood ratio test: χ^2 (2) = 9.61, p = .008; AIC = 25,449 vs. 25,454). No adjustments were made for multiple comparisons as all interaction terms were estimated within the same model.

SUPPLEMENTARY FIGURES

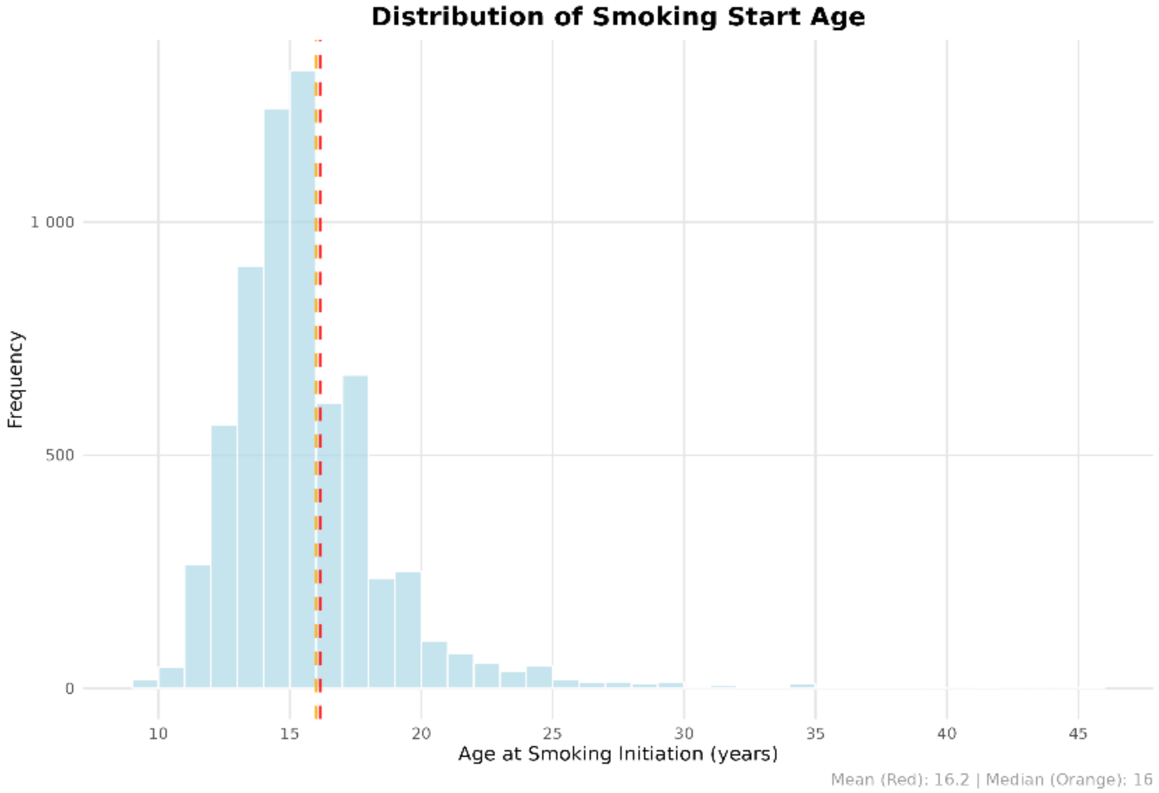

Supplementary Figure 1. **Distribution of age at smoking initiation among ever-smokers.** Age at smoking initiation among ever-smokers (*N* = 6,557). The distribution shows a pronounced peak in adolescence, with a mean age of 16.2 years (SD = 3.08, median = 16, range = 9–46). The red dashed line indicates the mean, and the orange dashed line indicates the median. The majority of smoking initiation occurred during adolescence, with only a small minority initiating after age 25.

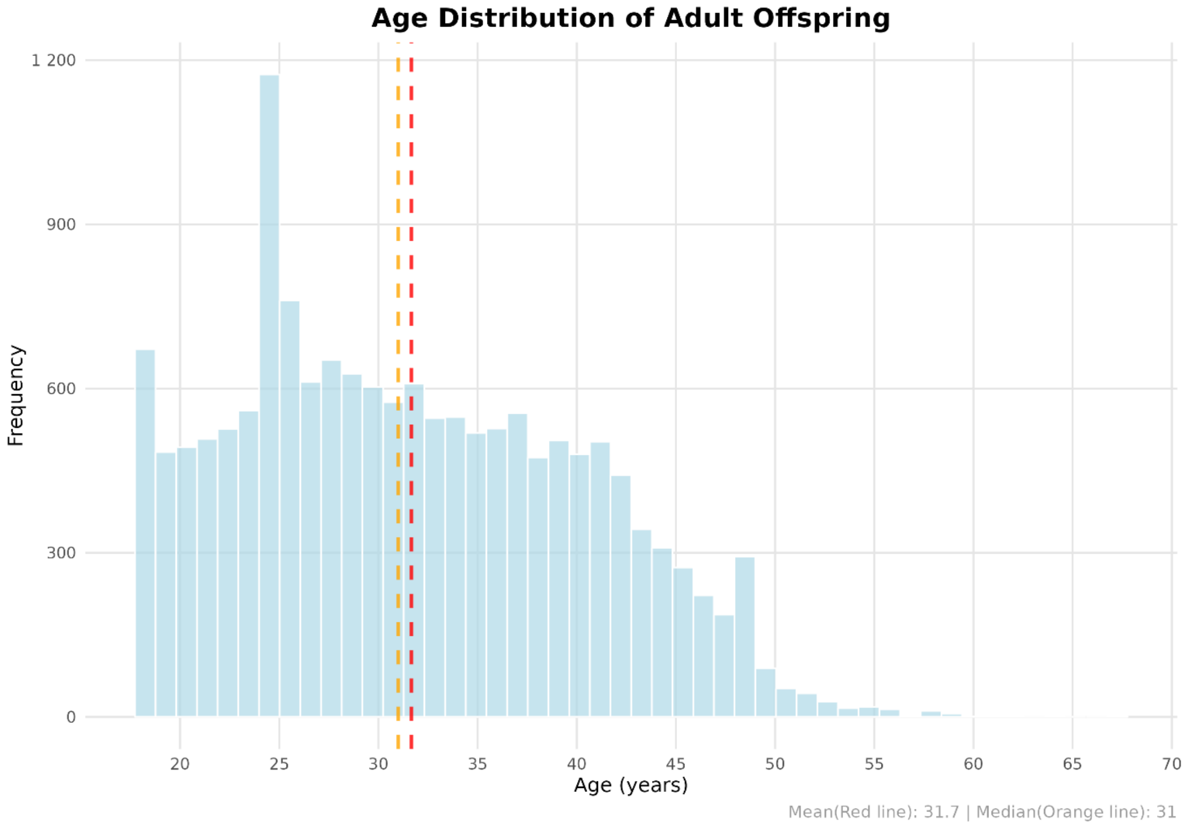

Supplementary Figure 2. **Age distribution of adult offspring at baseline assessment.** Age distribution of the adult offspring sample at baseline (*N* = 15,871). The sample represents a population-based adult cohort with a mean age of 31.7 years (SD = 8.61, range = 18–67). The red dashed line indicates the mean, and the orange dashed line indicates the median.

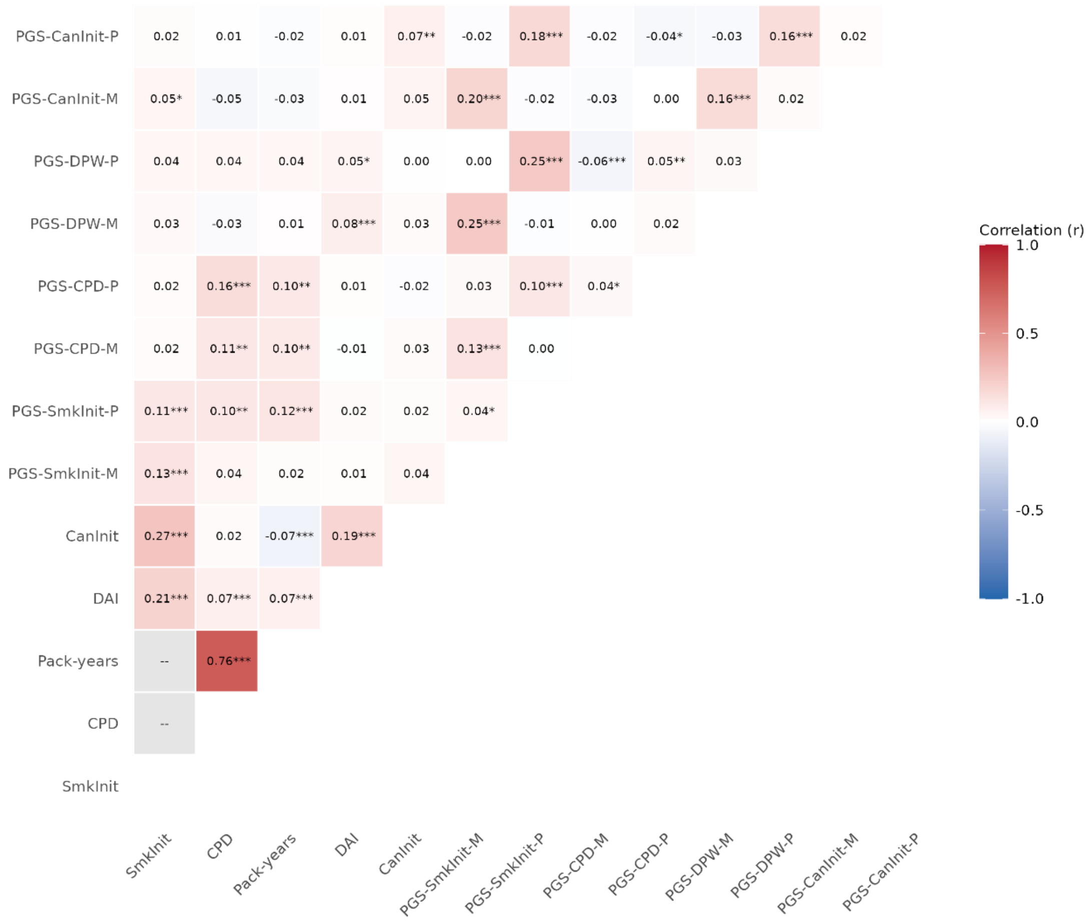

Supplementary Figure 3. Correlation heatmap of parental polygenic scores (PGS) and offspring substance use outcomes in the Lifelines cohort. Lower triangle displays correlation coefficients among offspring substance use phenotypes and parental PGS. Pearson's correlation was used for continuous–continuous variable correlations, point-biserial correlation for binary-continuous correlations, and the phi coefficient for binary-binary correlations. SmkInit = smoking initiation; CPD = cigarettes per day; DAI = daily alcohol intake; CanInit = cannabis initiation; M = mother; P = father. CPD and pack-years are defined only for ever-smokers; correlations with SmkInit are not estimable (shown as '--'). Offspring substance use sample sizes ranged from 5,969 to 15,937. Polygenic scores (PGS) were computed as the sum of transmitted and non-transmitted PGS for each parent using the complete trio sample (N = 3,267). Color scale indicates correlation strength from negative (blue) to positive (red). Significance levels: *$p < .05$; **$p < .01$; ***$p < .001$ (two-sided tests).

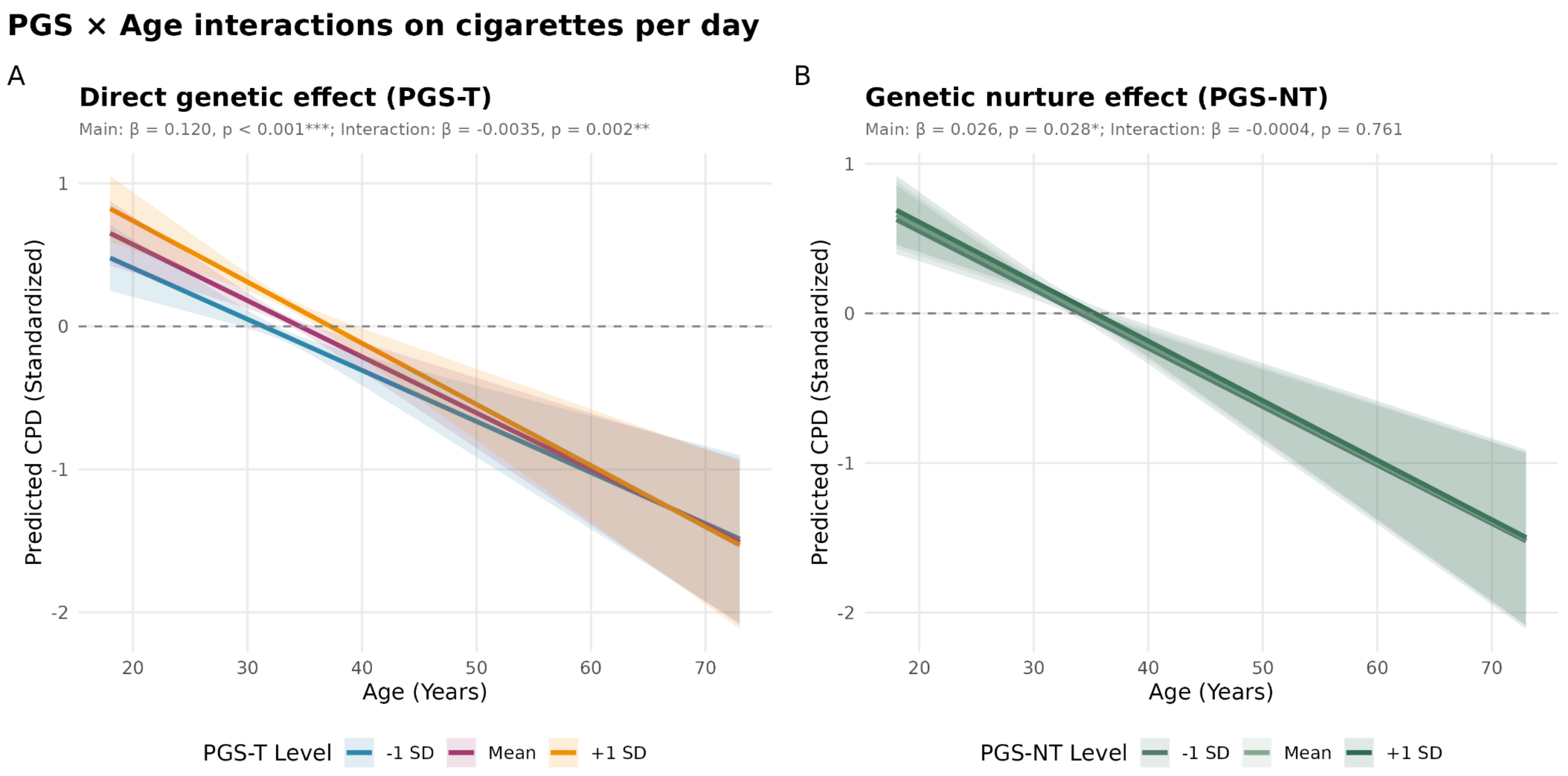

Supplementary Figure 4. Polygenic score × Age interactions on cigarettes per day. Predicted standardized cigarettes per day (CPD) across age at three levels of polygenic scores for CPD (−1 SD, mean, +1 SD). Shaded areas represent 95% confidence intervals. **A. Direct genetic effect**. The significant PGS_{T_CPD} × Age interaction (β = -.0035, p = .002) indicates that the effect of transmitted polygenic scores on CPD attenuates with age, as shown by the converging trajectories. **B. Genetic nurture effect.** The non-significant PGS_{NT_CPD} × Age interaction (β = -.0004, p = .761) indicates that genetic nurture effects remain stable across adulthood, as shown by the parallel trajectories. All models adjusted for sex, birth year, and measurement wave.

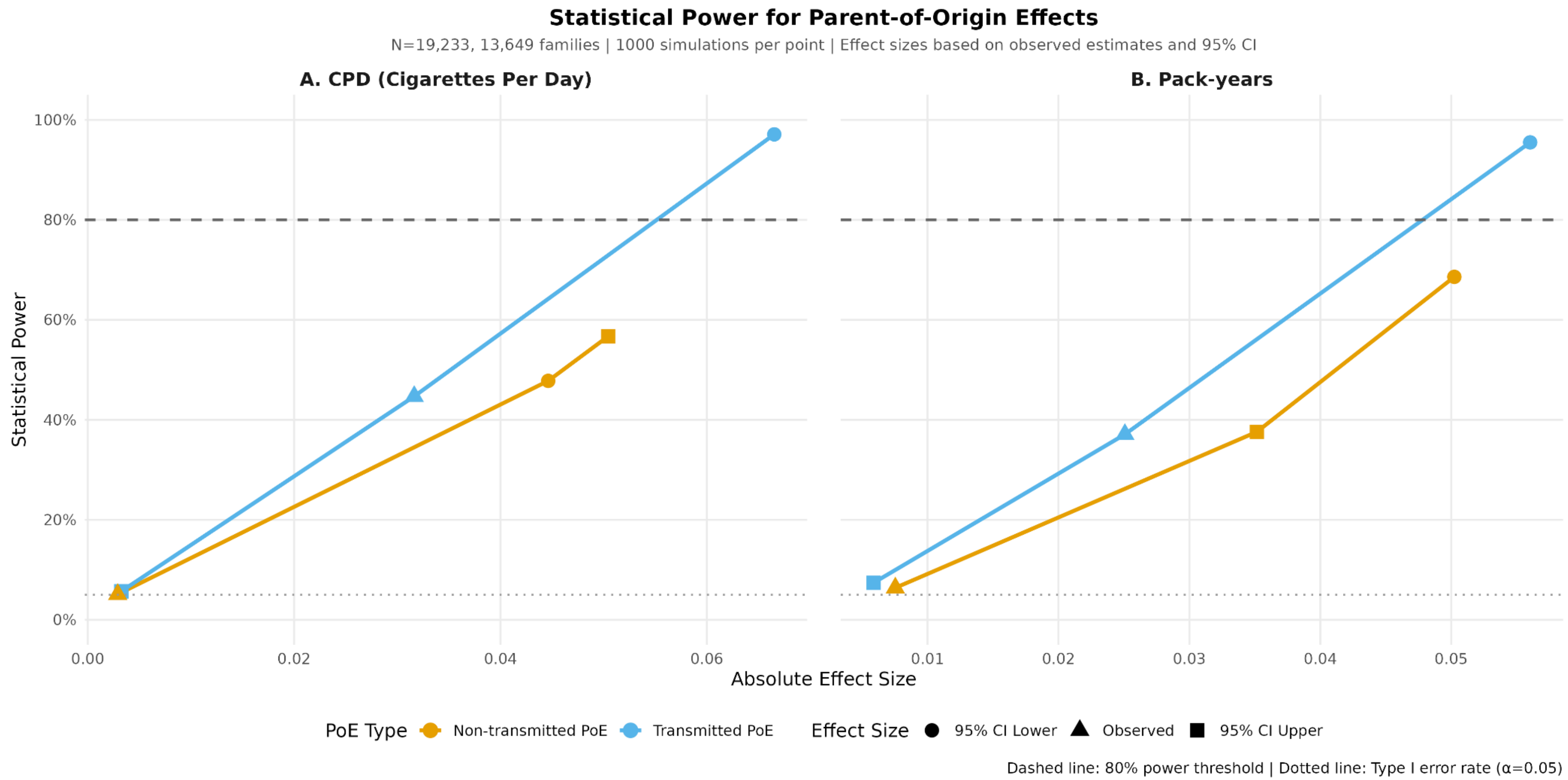

Supplementary Figure 5. Statistical power for parent-of-origin effects on smoking quantity. Monte Carlo simulations (1,000 replications per effect size) evaluated statistical power to detect transmitted (blue) and non-transmitted (orange) parent-of-origin effects across plausible effect sizes spanning the observed estimates and 95% confidence intervals. Panel A shows results for cigarettes per day (CPD); Panel B shows results for pack-years. The x-axis represents effect sizes (*Δβ*) for the parent-of-origin contrast (maternal minus paternal). Markers indicate effect sizes corresponding to: 95% CI lower bound (circle), observed estimate (triangle), and 95% CI upper bound (square). The dashed horizontal line marks the conventional 80% power threshold; the dotted horizontal line marks the Type I error rate (α = 0.05).
