# Supplementary material for: Genetic nurture in intergenerational transmission of substance use
Source: Nat Commun. 2026 Mar 26;17:4446. doi: 10.1038/s41467-026-71175-8 (PMC13183951; doi:10.1038/s41467-026-71175-8)
Supplement: Supplementary file 3 — Reporting Summary [file 41467_2026_71175_MOESM3_ESM.pdf]

nature portfolio

Corresponding author(s): Mannan Luo

Last updated by author(s): Feb 5, 2026

Reporting Summary

Nature Portfolio wishes to improve the reproducibility of the work that we publish. This form provides structure for consistency and transparency in reporting. For further information on Nature Portfolio policies, see our Editorial Policies and the Editorial Policy Checklist.

Statistics

For all statistical analyses, confirm that the following items are present in the figure legend, table legend, main text, or Methods section.

n/a	Confirmed	
☐	☒	The exact sample size (*n*) for each experimental group/condition, given as a discrete number and unit of measurement
☐	☒	A statement on whether measurements were taken from distinct samples or whether the same sample was measured repeatedly
☐	☒	The statistical test(s) used AND whether they are one- or two-sided<br>*Only common tests should be described solely by name; describe more complex techniques in the Methods section.*
☐	☒	A description of all covariates tested
☐	☒	A description of any assumptions or corrections, such as tests of normality and adjustment for multiple comparisons
☐	☒	A full description of the statistical parameters including central tendency (e.g. means) or other basic estimates (e.g. regression coefficient) AND variation (e.g. standard deviation) or associated estimates of uncertainty (e.g. confidence intervals)
☐	☒	For null hypothesis testing, the test statistic (e.g. *F*, *t*, *r*) with confidence intervals, effect sizes, degrees of freedom and *P* value noted<br>*Give P values as exact values whenever suitable.*
☒	☐	For Bayesian analysis, information on the choice of priors and Markov chain Monte Carlo settings
☒	☐	For hierarchical and complex designs, identification of the appropriate level for tests and full reporting of outcomes
☐	☒	Estimates of effect sizes (e.g. Cohen's *d*, Pearson's *r*), indicating how they were calculated

Our web collection on statistics for biologists contains articles on many of the points above.

Software and code

Policy information about availability of computer code

Data collection	No software was used for data collection.
Data analysis	Statistical analyses were performed in R (version 4.2.1). Transmitted and non-transmitted alleles were differentiated based on a validated haplotype-based approach (HINTA; Trindade Pons et al., 2024). Polygenic scores were constructed using LDpred2 (bigsnpr, version 1.12.21. Mixed-effects linear regression (lmerTest, version 3.1-3) was used for continuous outcomes and mixed-effects logistic regression (GLMMadaptive, version 0.8-5) for dichotomous outcomes. Parent-of-origin, mediation, and longitudinal models were implemented using structural equation modeling in Lavaan (version 0.6-12).<br>The analysis code is available at the GitHub: https://github.com/mannanluo/GeneticNurture_SU.

For manuscripts utilizing custom algorithms or software that are central to the research but not yet described in published literature, software must be made available to editors and reviewers. We strongly encourage code deposition in a community repository (e.g. GitHub). See the Nature Portfolio guidelines for submitting code & software for further information.

Data

Policy information about availability of data

All manuscripts must include a data availability statement. This statement should provide the following information, where applicable:

- Accession codes, unique identifiers, or web links for publicly available datasets
- A description of any restrictions on data availability
- For clinical datasets or third party data, please ensure that the statement adheres to our policy

DATA AVAILABILITY
Individual-level data from the Lifelines Cohort Study are available under restricted access due to ethical requirements and privacy regulations protecting participant confidentiality. Researchers can apply to use the Lifelines data through the online application system. More information about how to request Lifelines data and the conditions of use can be found on their website (https://www.lifelines.nl/researcher/how-to-apply). GWAS summary statistics used to construct polygenic scores were obtained from the following publicly available sources: smoking initiation, cigarettes per day and drinks per week from the GWAS & Sequencing Consortium of Alcohol and Nicotine use (GSCAN; https://doi.org/10.13020/przg-dp88); substance use disorder from the Psychiatric Genomics Consortium (https://www.med.unc.edu/pgc/).

Research involving human participants, their data, or biological material

Policy information about studies with human participants or human data. See also policy information about sex, gender (identity/presentation), and sexual orientation and race, ethnicity and racism.

Reporting on sex and gender	Biological sex (female/male) assigned at birth was included as a covariate in all primary models; the analytic sample was 61.9% female. Parent-of-origin effects were evaluated by estimating maternal and paternal transmitted and non-transmitted polygenic scores separately using structural equation modeling (SEM). We additionally conducted offspring sex-stratified analyses using multi-group SEM to assess whether mediation pathways through parental smoking differed between daughters and sons.
Reporting on race, ethnicity, or other socially relevant groupings	Race and ethnicity were not used as study variables. Analyses were restricted to participants of European ancestry because the Lifelines cohort is predominantly of European ancestry (around 95%, Zhang et al., 2021) and the polygenic scores were derived from GWAS conducted in European-ancestry samples.
Population characteristics	The analytic sample comprised genotyped European-ancestry participants with at least one genotyped parent in Lifelines. Detailed participant characteristics (age, sex, and relevant behavioral measures) are provided in Table 1.
Recruitment	In the LifeLines Cohort Study, a recruitment strategy was adopted that aimed to include three generations of participants. Firstly, all GPs in the three northern provinces of the Netherlands were invited to participate and asked to invite their registered patients aged 25–49 years. Patients who were unable to read Dutch or who had limited life expectancy due to severe illness were excluded by the GP and not invited for participation. Participants who gave written informed consent were included as the“index population”. Subsequently, all persons in the index population were asked to indicate whether family members (partner, parents, parents-in-law, and children) could be invited and to provide their contact details. Family members were invited by Lifelines; those who gave their informed consent were included in the study as “family member”. Furthermore, persons aged 18 years and older could participate in this study through“self-registration” via the Lifelines website. These self-registrants were also asked to invite family members as outlined above. Lifelines aimed to include three generations of participants, but individuals who had no family member participating in the study were not excluded (Klijs et al. 2015).
Ethics oversight	The Lifelines protocol has been approved by the UMCG Medical ethical committee under number 2007/152.

Note that full information on the approval of the study protocol must also be provided in the manuscript.

Field-specific reporting

Please select the one below that is the best fit for your research. If you are not sure, read the appropriate sections before making your selection.

☐ Life sciences ☒ Behavioural & social sciences ☐ Ecological, evolutionary & environmental sciences

For a reference copy of the document with all sections, see nature.com/documents/nr-reporting-summary-flat.pdf

Behavioural & social sciences study design

All studies must disclose on these points even when the disclosure is negative.

Study description	Quantitative study
Research sample	We utilized data from 19,233 genotyped adult offspring with at least one genotyped parent from the Dutch population-based Lifelines cohort, including 15,966 parent–offspring pairs and 3,267 mother–father–offspring trios. Of these, up to 15,863 participants (mean age at baseline = 31.66 years; 61.9% female), comprising 13,411 pairs and 2,452 trios with available substance use data, completed baseline assessments of tobacco and alcohol use (2006–2013) and wave 2 assessments of cannabis use (2014–2017). Descriptive statistics are presented in Table 1. Lifelines is a general population cohort and broadly representative for the adult

	population of the north of the Netherlands (Klijs et al., 2015).
Sampling strategy	We used the largest available sample of genotyped parent–offspring pairs and trios in Lifelines with substance use data. This sample was chosen because genotyped parents and offspring enable separation of transmitted and non-transmitted genetic variants, while accounting for different family structure and maximizing power by including both pairs and trios.
Data collection	Substance use phenotypes were obtained from the Lifelines study using standardized self-report questionnaires administered by the cohort at scheduled assessments. Tobacco outcomes included smoking initiation and smoking quantity (cigarettes per day and pack-years). Alcohol outcomes were derived from questionnaire-based consumption measures (e.g., daily alcohol intake). Cannabis use was assessed at wave 2 using self-report questions on lifetime use. Data were collected by the Lifelines research staff as part of the cohort protocol; the present research team was not present during participant assessments. This study is a secondary analysis of an existing observational dataset with no experimental manipulation; therefore, researcher blinding to experimental condition was not applicable.
Timing	The Lifelines baseline assessment was conducted between 2006 and 2013, followed by wave 2 (2014–2017) and wave 3 (2019–2023) (with a gap between wave 2 and wave 3). Tobacco and alcohol use were assessed at baseline, cannabis use was assessed at wave 2, and current cigarettes per day (CPD) was assessed repeatedly across all three waves.
Data exclusions	Patients who were unable to read Dutch or who had limited life expectancy due to severe illness were excluded by the GP and not invited for participation. Individuals of non-European genetic ancestry were excluded because the PGS weights were based on European-ancestry GWAS, and applying these scores to other ancestry groups can lead to reduced predictive performance and biased effect estimates.
Non-participation	Not applicable. The study is based on already collected data.
Randomization	Not applicable. This was an observational study, no randomization was performed.

Reporting for specific materials, systems and methods

We require information from authors about some types of materials, experimental systems and methods used in many studies. Here, indicate whether each material, system or method listed is relevant to your study. If you are not sure if a list item applies to your research, read the appropriate section before selecting a response.

Materials & experimental systems

n/a	Involved in the study
☒	☐ Antibodies
☒	☐ Eukaryotic cell lines
☒	☐ Palaeontology and archaeology
☒	☐ Animals and other organisms
☒	☐ Clinical data
☒	☐ Dual use research of concern
☒	☐ Plants

Methods

n/a	Involved in the study
☒	☐ ChIP-seq
☒	☐ Flow cytometry
☒	☐ MRI-based neuroimaging

Plants

Seed stocks	NA
Novel plant genotypes	NA
Authentication	NA
